# The "Rebirth" of the Right Anterolateral Thoracotomy Approach in Cardiac Surgery

**DOI:** 10.21470/1678-9741-2018-0602

**Published:** 2018

**Authors:** Domingo M. Braile, Paulo Roberto B. Evora

**Affiliations:** 1 Editor-in-Chief - BJCVS. Faculdade de Medicina de São José do Rio Preto (FAMERP), São José do Rio Preto, SP, Brazil and Universidade de Campinas (UNICAMP), Campinas, SP, Brazil.; 2 Editor-in-Chief Interim - BJCVS Faculdade de Medicina de Ribeirão Preto da Universidade de São Paulo (FMRP-USP), Ribeirão Preto, SP, Brazil

## BJCVS Highlight

**Fig. 1:**
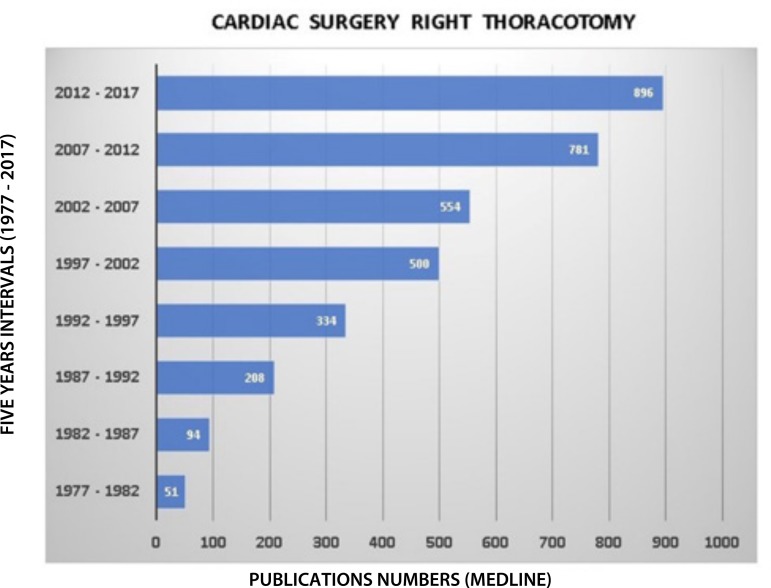
Five-year distribution of publications over thirty years (1977-2017), based on a free MEDLINE database search ("Right thoracotomy and cardiac surgery").

The right anterolateral thoracotomy approach was routinely used for atrial septal, mitral valve and tricuspid valve surgeries in the 1970's and 1980's. Currently, it is considered "as a wellaccepted technique"^[1]^, despite the almost universal acceptance of conventional sternotomy. The freely MEDLINE search (Figure 1) illustrates the "rebirth" of right thoracotomy and is evident that several groups have been reported their experiences with this surgical approach. The right anterolateral thoracotomy approach has advantages compared to the standard median sternotomy: 1) The surgical scar is invisible under the breast; 2) The exposition of intracardiac structures is excellent; 3) The intraoperative complication rate is almost zero, and; 4) Phrenic nerve damage, which is primarily attributed to right anterolateral thoracotomy, is uncommon. Also, in addition to the operative details above, blood transfusion and chest drainage, intubation time, intensive care unit (ICU) and hospital stay, are not associated with increased postoperative complications.

Finally, recent publications have to emphasize the right anterolateral thoracotomy as an attractive alternative to repeat sternotomy for patients undergoing redo mitral and tricuspid valve repair. The anterior thoracotomy has seen a resurgence with the recent trends towards minimally invasive cardiac surgical techniques. We agree with this alternative, and our preference includes mitral valve exposition by right atriotomy, followed by an atrial septostomy.

## Articles in this Issue

This issue of BJCVS presents a blind peer-reviewed selection of 14 articles that will surely please your readers. The articles are about: coronary artery disease (4 papers); heart valve disease (2 papers), congenital heart disease (1 paper); aortic aneurysms (2 papers); cardiac electrical stimulation (1 paper); selected case report (1 paper); and perioperative subjects (3 papers).

The present edition has an essential historical background. For the first time, the number of international contributions (9 articles/64.3%) exceeded the number of Brazilian articles (5 articles/35.7%). Most publications come from Eastern countries (Turkey and China). We hope that this trend will continue to mean an internationalization, a more significant number of citations and, consequently, a greater impact factor. Alongside this trend, Brazilian surgeons and researchers need to engage in publications based on quantity/quality binomial.

**Domingo M. Braile** ^1^Editor-in-Chief - BJCVS Faculdade de Medicina de São
José do Rio Preto (FAMERP), São José do Rio Preto, SP, Brazil
and Universidade de Campinas (UNICAMP), Campinas, SP, Brazil.**Paulo Roberto B. Evora** ^2^Editor-in-Chief Interim - BJCVS Faculdade de Medicina de
Ribeirão Preto da Universidade de São Paulo (FMRP-USP),
Ribeirão Preto, SP, Brazil
